# Does Environmental, Social, and Governance Drive the Sustainability of Multinational Corporation’s Subsidiaries? Evidence From Korea

**DOI:** 10.3389/fpsyg.2022.899936

**Published:** 2022-05-13

**Authors:** Jangsoon Kim, Eunho Cho, Collins E. Okafor, Donseung Choi

**Affiliations:** ^1^Department of Business, Sogang University, Seoul, South Korea; ^2^Department of Accounting and Finance, North Carolina A&T State University, Greensboro, NC, United States; ^3^Department of International Trade, Andong National University, Andong, South Korea

**Keywords:** ESG, sustainability, MNC, subsidiary, market-oriented organizational culture, moderating effect

## Abstract

We examined the relations between environmental, social, and governance (ESG) activities and the performance of subsidiaries of multinational corporations (MNCs). We further investigated the moderating effect of market-oriented organizational culture on the relationship between ESG and performance. Employing generalized least square regression analysis using survey data, we show that ESG activities of MNC subsidiaries are positively associated with financial and non-financial performance. These results suggest that ESG improves the financial and non-financial performance of subsidiaries. The test for the moderating effect of the market-oriented organizational culture shows that it weakens the positive relationship between ESG activities and financial performance. This could be due to the incongruous nature of the short-term focus of a market-oriented organizational culture versus the long-term orientation of the sustainability of ESG activities.

**JEL Code:** D64, G32, M14

## Introduction

This study examines the relationship between environmental, social, and governance (ESG) activities and the performance of subsidiaries of multinational corporations (MNCs). Furthermore, we investigate the moderating effect of market-oriented organizational culture on the relationship between ESG and performance. The growing global campaign for ESG makes it increasingly necessary to understand better the factors that hamper or facilitate its practice across firms. Firms’ interest in ESG is not just voluntary. It is also primarily driven by an increase in the number of investors that sought after ESG-based assets and the pressure from civic groups and international organizations.

Environmental, social, and governance could also provide a cushion for firms during times of economic downturn. Gilllan et al. (2021) posit that high ESG firms show performance resilience during an economic crisis by harnessing value through financial figures and non-financial avenues.

In particular, after the 2008 global financial crisis, reflection on neoliberalism, shareholder capitalism, and resistance to deepening economic inequality acted as critical environmental factors. Therefore, in the context of “the double power principle,” the market forces contributed directly and indirectly to the proliferation of interest in ESG. Discussed as an alternative to the recovery to a moral economy and shareholder-first principle, ESG focuses on sustainability as a core value by linking economic and social values.

The concept of sustainability was first discussed at the World Commission on Environment and Development (WCED) in 1987. WCED, in its 1987 report entitled “Our common future,” defined sustainable development as development that meets the needs of the present without sacrificing the ability of future generations to meet their own needs. Durand et al. (2019) define sustainability as the possibility of meeting immediate short-term needs while meeting the requirements for securing the future. Consequently, companies are now publishing sustainability reports according to the guidelines of the 2016 Global Report Initiative (GRI). Since the term ESG was first introduced in the UN Environment Program Financial Initiative in 2003, the UN Principles for Responsible Investment were presented in 2006, focusing on ESG indicators.

As a result, MNCs have strengthened their social, ethical, and environmental responsibilities in the global market. Carroll (2004) predicts that the pressure on firms to protect the environment, promote social responsibility, and practice transparent management will increase because corporate social responsibility (CSR) activities have become a global mainstay. Therefore, it is crucial and time-relevant to investigate whether the headquarters of an MNC and its subsidiaries are fulfilling their responsibilities as global market leaders (Luo, 2006). In this context, this study empirically analyzes the relationship between ESG and the performance of subsidiaries of MNCs located in Korea.

The resource-based theory could explain how a subsidiary of an MNC generates financial performance through ESG activities. Subsidiaries strategically and preemptively conduct ESG activities from a long-term perspective to solve local problems and gain a sustainable competitive advantage. The external factors that cause subsidiaries to engage in ESG activities can be examined from stakeholder theory and institutional duality. The resource-based theory focuses on the internal factors of a company to build a competitive advantage. In contrast, the stakeholder theory and institutional duality are related to the company’s external factors. As with the general discussion of ESG, subsidiaries of MNCs conduct ESG activities to meet the expectations and needs of local stakeholders, which can positively impact business performance in the long run. Institutional duality can explain the ESG activities carried out in reconciling the different interests to meet the local needs that the subsidiaries face with the strategic guideline of the global standard established at the headquarters level.

This study conducted an empirical analysis of data collected through a questionnaire survey on subsidiaries of MNCs located in Korea from December 1, 2021, to February 28, 2022. Respondents to the survey were male (50.7%) and female (49.3%). The average age of the respondents was 39.53, and the average tenure was 8.5 years. Before sending the survey questionnaire to the respondents *via* email, a phone call was made first to solicit their interest and ensure participation and completion rate.

The main results and implications of this study are as follows. First, the MNCs’ ESG activities are positively associated with the financial and non-financial performance of subsidiaries of MNCs. Second, a firm’s market-oriented organizational culture weakens the positive relationship between ESG and performance. Lastly, we find that market-oriented organizational culture did not play a moderating role in the relationship between ESG and non-financial performance.

The contributions of this study are as follows. First, to the best of our knowledge, this study is the first to analyze the relationship between ESG and performance for subsidiaries of MNCs using survey data. Second, this study divides non-financial and financial performance to examine various aspects of business performance. Third, we investigated the moderating effect of market-oriented organizational culture on the relationship between ESG and the performance of subsidiaries of MNCs.

The rest of this study is organized as follows. The following part explains the theoretical background of how ESG can affect organizational performance from the perspective of MNCs. Then, we propose hypotheses on the relationship between variables. Next, we introduce the data and analysis methods used in this study and present the empirical results. Finally, we discuss the conclusions and practical implications.

## Theoretical Background

### Environmental, Social, and Governance

Firms implement ESG policies and diligently enforce its practice when they desire to achieve sustainable management. ESG consists of environmental, social responsibility, and transparent management (Friede et al., 2015). ESG management can be seen as slightly different from CSR. It focuses on firms that show resilience during times of crisis by demonstrating sustainability and reflecting value from the financial figures and non-financial factors (Gilllan et al., 2021).

Despite the aforementioned differences, it would be false-hearted to deny that interest in ESG management originated from prior research on CSR. Instead, ESG can be viewed as a concept developed from CSR rather than a completely different concept from CSR. In other words, it is reasonable to view ESG as a concept, with some additions or adjustments, that supplements CSR. In practice, it is recognized as an essential toolbox that helps firms attain sustainability. Previous studies on sustainability management use ESG and CSR similarly in research models can be seen as supporting the supplementing-relationship argument. McWilliams and Siegel (2001) define CSR activities as corporate activities aimed at creating a better society beyond meeting the requirements set by laws and regulations. Eccles et al. (2014) assert that it is those activities that firms undertake which determine their future performance through consideration of the environment, society, and governance.

Friede et al. (2015) examine existing studies on CSR from the viewpoint of emphasizing each element of the **e**nvironment (E), **s**ocial responsibility (S), and transparent management through a **g**overnance system (G). A closer examination of CSR studies shows that the focus is on the environment and social responsibility. In conclusion, the ultimate goal of ESG, like CSR, is to pursue sustainable growth from a long-term perspective and to have a positive impact on social benefits. However, ESG is different from CSR in that ESG is based on the triple bottom line (TBL) to evaluate the level of sustainable management from an investor’s point of view. Therefore, we can argue that the three clear concepts of ESG emphasized in the study of Eccles et al. (2014) can be correctly implemented only when the part corresponding to G is reflected in the research model (Friede et al., 2015).

### Environmental, Social, and Governance in Multinational Corporation Perspective

We need to look at the research on ESG of MNCs based on the following three theoretical contents. First, overseas subsidiaries of MNCs have to survive by solving local problems from a resource-based point of view and carrying out activities to gain a competitive advantage in the local market. In this process, local subsidiaries face the challenge of overcoming resource limitations as outsiders who do not have sufficient external justification (Zaheer and Mosakowski, 1997; Kostova and Zaheer, 1999). MNCs can implement environmental, social responsibility, and ethical management activities to secure assets to solve the problem of liability of foreignness that the subsidiaries have to pay to acquire information or the problem of external legitimacy lacking in the subsidiaries (Zaheer, 1995; Nachum, 2003). In addition, subsidiaries entering the growth phase pursue opportunities and engage in value-enhancing activities that continuously strengthen the company’s competitiveness. Thus it is imperative that the subsidiaries of MNCs actively engage in activities related to the local environment, social responsibility, and ethical management (Meyer, 2017).

Second, subsidiaries of MNCs will conduct ESG management to meet the expectations and needs of local stakeholders from the point of view of CSR. MNCs entering developing countries, including emerging markets, can lay the groundwork for sustainable growth with their subsidiaries and related stakeholders by simultaneously meeting environmental, social, and local economic needs (Jamali et al., 2011). When an MNC operates in a host country with a lower level of economic development than its home country, activities that address local stakeholders’ economic, environmental, and social needs are the social responsibility implicitly required of subsidiaries (Hart and Christensen, 2002). The degree of responsibility differs based on the host country. Suppose the level of development is lower than that of the home country. There may be environmental specificities inherent in social problems, such as a decrease in education level, unemployment, and poverty.

Conversely, if the economic level of the host country is higher than that of the home country, the overseas subsidiary needs to demonstrate ESG activities that comply with global standards. Since MNCs must operate in different business environments between countries, their subsidiaries cannot help but be under the influence of local stakeholders. Therefore, the sensitivity to environmental, social responsibility, and ethical management activities experienced by subsidiaries in the local market will be high. Consequently, it is essential to carry out ESG activities for subsidiaries of MNCs in developing countries. It enables innovation at the level of the corporate value chain and provides answers to sustainability management (London and Hart, 2004).

Finally, subsidiaries of MNCs conduct ESG management from the perspective of institutional duality. Overseas subsidiaries have to reconcile their various interests in conducting global management. As a preemptive measure, they can be under pressure to promote and engage in ESG activities locally. Environmental and social responsibility activities directed by the head office have to take into account the strategic choice of the parent company, internal processes, and stakeholder groups while also taking into account the impact of regulations and norms at various levels in the host country (Zhao et al., 2014). This also has to do with how much autonomy a subsidiary has from its headquarters. When reviewed comprehensively, the institutional duality of MNCs can be said to be an issue that is caused by the characteristics of subsidiaries internally as well as in various external environments. In other words, MNCs inevitably face various institutional factors when managing their ESG activities, ranging from local optimization or global market-leading standardization to host country-centric or host-country-centric issues. Therefore, some of the ESG activities at the headquarters level of MNCs are transferred to overseas subsidiaries. However, some ESG activities are initiated at the local subsidiary level.

## Hypothesis Development

### Environmental, Social, and Governance, Performance, and Sustainability

Recently, some studies (Dyllick and Hockerts, 2002; Springett, 2003) highlighted the concept of sustainability that satisfies the needs of shareholders and stakeholders without compromising the company’s capabilities. Sustainability is a management activity that enhances the organization’s value by promoting communication with the company and stakeholders. Sustainability management can also be ideal for a company that seeks to maximize shareholder value by increasing corporate profits in alignment with the expanding stakeholders’ specific interests (Edmans, 2021). From this perspective, our study uses subsidiaries’ financial and non-financial performance as a proxy for sustainability. Research on sustainability generally analyzes the impact of ESG activities on performance, consisting of financial and non-financial performance, represented by shareholder value (Waddock and Graves, 1997; Margolis and Walsh, 2003; Orlitzky et al., 2003; Edmans, 2011).

The results of studies analyzing the effects of ESG on performance do not converge in one direction. There are mixed results. The findings vary from no significant relationship between two variables to a positive association or negative relationship. Some report a U-shaped or inverse U-shaped relationship (Teoh et al., 1999; Wright and Ferris, 1997; McWilliams and Siegel, 2000; Arora and Dharwadkar, 2011). Nonetheless, most studies report that corporate ESG activities positively affect firm performance (Waddock and Graves, 1997; Barnett, 2007; Wang and Qian, 2011; Aguinis and Glavas, 2012).

Waddock and Graves (1997) argued that non-financial performance could positively affect financial performance, such as stock price or profit margin, leading to a sustainable virtuous cycle. Also, research results showed that such a virtuous cycle of performance is observed better in MNCs that conduct business as subsidiaries in other countries. They have more free resources and stronger stakeholder influence than smaller corporations (Barnett, 2007).

### Environmental, Social, and Governance and Financial Performance

If most studies had shown that CSR activities hurt corporate performance, it would have been for the campaign for CSR to gain any traction. Most empirical findings support CSR’s value-enhancing arguments, translating to a positive effect on firm performance. Carroll (1979) classified the beneficiaries of economic and philanthropic responsibilities tied to CSR activities into shareholders and stakeholders, respectively, and argued that CSR could positively affect financial performance. In addition, several studies have concluded that CSR has a positive effect on corporate performance (Waddock and Graves, 1997; Orlitzky et al., 2003; Barnett, 2007; Wang and Qian, 2011; Aguinis and Glavas, 2012; Cho and Tsang, 2020).

Environmental, social, and governance, which has a more active meaning than CSR, is highly likely to positively affect firm performance (Friede et al., 2015). ESG activities of subsidiaries meet the expectations of local stakeholders by meeting the economic and social needs of the local country, which can give the impression that the company is active in the localization (Jamali, 2007). Local stakeholders impressed and satisfied by the subsidiary’s ESG activities will be more willing to provide more valuable resources that contribute to the growth and success of the subsidiary’s business (Peng and Luo, 2000). Subsidiaries that are more active in ESG engagements with local stakeholders could reduce operating costs and even save money when acquiring the necessary resources needed for their business (McWilliams and Siegel, 2011). In addition, resources provided by local stakeholders increase the ability of subsidiaries to adapt to local conditions and become a driving force for performance improvement (Wang et al., 2008). Therefore, we can expect that the ESG management of subsidiaries will positively affect financial performance. Based on the above discussion, we propose the following hypotheses.

**Hypothesis 1:** The ESG activities of a subsidiary of MNCs can lead to higher financial performance.

### Environmental, Social, and Governance and Non-financial Performance

As discussed above, the subsidiaries of MNCs can conduct ESG activities to create and sustain a competitive advantage in the local market based on the resource-based theory. The competitiveness of these companies is closely related to financial performance and non-financial performance. If a subsidiary concentrates too much on financial profits, its core business may suffer long-term, declining productivity. Expansion into a new market unrelated to an existing business may destroy social value, which corresponds to non-financial performance, and endanger the growth of the local community and the survival of the company (Moeller et al., 2005). However, suppose a subsidiary implements a strategy that considers the environment, consumers, employees, financial supporters, suppliers, regulators, and the community. In that case, it can create social value with strong externalities (Jensen, 2002). Jamali (2010) argued that the activities of a subsidiary of MNCs paying attention to the education and safety issues of the local community and providing public infrastructure create social value. The actions of such subsidiaries can improve the corporate image (Tully and Winer, 2014). Therefore, subsidiaries conducting business in the local market may induce various externalities related to social responsibility (Jamali, 2008).

Drawing from the arguments, we posit that the local subsidiaries’ management of its ESG will positively affect non-financial performance, which is a social value, including the image of the subsidiary perceived by local stakeholders. Based on the above discussion, we establish the following hypothesis.

**Hypothesis 2:** The ESG activities of a subsidiary of MNCs can lead to higher non-financial performance.

### The Moderating Effect of Market-Oriented Organizational Culture

Each organization has its unique cultural characteristics. The extant research on organizational culture has focused on categorizing organizational culture, identifying common characteristics and culture-determining factors, explaining the characteristics of culture, and revealing organizational effectiveness (Denison and Mishra, 1995; Cameron and Quinn, 2011). Previous studies have also identified organizational cultures by classifying them based on organizational characteristics (Ketchen, Jr, Thomas and Snow, 1993). As part of this classification, Cameron and Quinn (2011) developed Quinn’s theoretical model published in 1988 to define organizational culture types as flexibility and discretion, stability and control, inward orientation, and integration. Organizational culture was classified based on four factors: (internal focus and integration) and external focus and differentiation. Our study examines the moderating effect of market-oriented organizational culture, which has elements of stability and control, external orientation, and discrimination in the relationship between ESG and performance. According to Cameron and Quinn (2011), organizational culture can be classified into clan, adhocracy, hierarchy, and market cultures. As such, there are various organizational cultures, but among them, the market-oriented organizational culture is selected as a moderating variable because our study is conducted on subsidiaries of MNCs. A market-oriented organizational culture based on competition may not be strategically aligned with ESG management focused on long-term sustainability in that it is oriented toward short-term performance.

A market-oriented organizational culture can be a type of organizational culture that emphasizes productivity in achieving organizational goals and performing tasks. Organizations with market-oriented culture value efficiency and reward for performance, from planning to achieving goals (Zammuto and Krakower, 1991). In particular, subsidiaries are highly likely to espouse the characteristics of a market-oriented organizational culture. They are organizations designed to produce tangible results from a local, short-term perspective from birth in corporate culture, goals, corporate structure, and decision-making (Hewett et al., 2003). This market-oriented organizational culture induces short-term performance-oriented activeness among members of the organization.

Subsidiaries’ market-oriented organizational culture fosters an atmosphere where subsidiaries focus on short-term outcomes rather than processes. Therefore, ESG is more likely to be perceived as a cost or unavoidable procedure rather than an immediate benefit to a subsidiary competing fiercely in the local market. However, ESG management focuses on long-term performance rather than short-term performance. Thus, ESG contrasts with the immediate results of a market-oriented organizational culture. This suggests that the subsidiary’s market-oriented organizational culture can become an obstacle to ESG management activities’ path to positive and tangible results. The strategic fit perspective argues that a company can improve its performance by enhancing the fit between its strategies and the particular environments where it operates (Katsikeas et al., 2006). Improving the fit between its strategic type and a firm’s characteristics is desirable because it produces a better performance (Miles and Snow, 1984; Porter, 1996; Zajac et al., 2000). Based on the above discussion, the following hypotheses were established.

**Hypothesis 3:** The market-oriented organizational culture of MNCs’ subsidiaries weakens the positive relationship between ESG and financial performance.

**Hypothesis 4:** The market-oriented organizational culture of MNCs’ subsidiaries weakens the positive relationship between ESG and non-financial performance.

## Research Design

**FIGURE 1:**
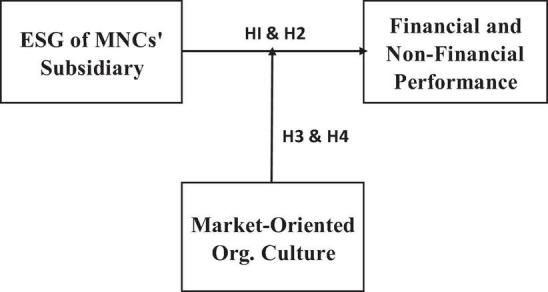
Research design.

Figure 1 illustrates the research model in our study.

We test the following Equation (1) to examine our hypotheses.


(1) $$\begin{array}{l} PERFORMANCE_{i,t}=\alpha_{0}+\alpha_{1}SUBSIDIARY^{\prime }S\_ESG_{i,t}\\ \;+\alpha_{2}MARKET-ORIENTED\_ORG\_CULTURE_{i,t}\\ \;+\alpha_{3}SUBSIDIARY^{\prime }S\_ESGXMARKET-ORIENTED\_ORG\_\\ \;CULTURE_{i,t}+\alpha_{4}SALES_{i,t}+\alpha_{5}PARENT\_EQUITY\_\\ \;SHARE_{,t}+\alpha_{6}SUBSIDIARY\_AGE_{,t}+\alpha_{7}HQ\_GLOBAL\_\\ \;ORIENTATION_{i,t}+\alpha_{8}LOCAL\_RESPONSIVENESS_{i,t}\\ \;+\alpha_{9}SUBSIDIARY^{\prime }S\_LEVEL\_OF\_LOCALIZATION_{i,t}\\ \;+\alpha_{10}SUBSIDIARY\_AUTONOMY_{i,t}\\ \;+\alpha_{11}INDUSTRY_{i,t}+\varepsilon_{i,t} \end{array}$$

**Table:** 

Variables	Description
*Dependent variables*	
*PERFORMANCE*	
*FINANCIAL_ PERFORMANCE*	= Mean value of the survey data of sales growth rate, market share, and operating profit
*NON-FINANCIAL_ PERFORMANCE*	= Mean value of the survey data of customer satisfaction, employee satisfaction, and reputation and image
*Independent variables*	
*SUBSIDIARY’S_ ESG*	= Environmental, social, and governance (ESG) of multinational corporation’s subsidiary, the mean value of environmental, social, and governance (Detailed items of ESG are shown in Appendix Table A1)
*MARKET-ORIENTED_ORG_ CULTURE*	= Subsidiary firm’s market-oriented organizational culture is measured by the extent to which (1) subsidiary is performance-oriented, (2) subsidiary’s leader is performance-oriented, (3) subsidiary values the progress of performance more than relationship, (4) subsidiary pursuit the mission accomplished ultimately, (5) subsidiary values the maximization of performance under a given circumstance, and (6) subsidiary values the competition with main competitors
*SALES*	= Natural logarithm of sales revenue for the fiscal year
*PARENT EQUITY_ SHARE*	= Parent firm’s equity share, a dummy variable taking 1 if the subsidiary’s type of foreign direct investment (FDI) is a greenfield investment and 0 if the subsidiary’s type of FDI is a brownfield investment
*SUBSIDIARY_ AGE*	= Subsidiary firm’s age
*HQ_ GLOBAL_ ORIENTATION*	= Headquarter firm’s global orientation measured by the extent to which our MNE-HQ concentrates on developing standardized products by considering the world market as a single unit, and our MNE-HQ provides the same advertisement, product, and design for targeting the transnational global market.
*LOCAL_ RESPONSIVENESS*	= Subsidiary firm’s local responsiveness is measured by the extent to which our MNE-HQ guides foreign subsidiaries to compete in their own markets, and our MNE-HQ corresponds to the needs of local markets.
*SUBSIDIARY’S_ LEVEL_ OF_ LOCALIZATION*	= Subsidiary firm’s level of localization measured by the extent to which the (1) product or service provided by the subsidiary, (2) research and development or marketing of subsidiary, (3) organization structure or operation of the subsidiary, (4) HR control (employing, development, etc.) of the subsidiary in the local market takes great importance of local trait than of head office’s standard procedure.
*SUBSIDIARY_ AUTONOMY*	= Subsidiary firm’s autonomy measured by the extent to which subsidiary’s decision making for (1) developing or introducing a new product in the local market, (2) expanding or reducing manufacturing facilities in the local market, (3) establishing or executing of budget in the local market, and (4) administrators (employment, expatriate, promotion) in the local market.
*INDUSTRY*	= Industry dummy taking 1 if the firms belong to the Manufacturing, 0 otherwise.

### Data

We obtained data through a survey from December 1, 2021, to February 28, 2022. The firms listed in the directory of foreign-invested enterprises operating in Korea compiled by the Investment Notification Statistics Center (INSC) of Korea were used as the sample of our model. INSC’s dataset provides the contact information of the subsidiaries of MNCs that have entered Korea. We focused on MNC subsidiaries in Korea, investigating their ESG and firm performance. South Korea has recorded substantial growth in the past few decades, supported by the rapid international investment from MNCs. As the economy of Korea entered an advanced level, the management of MNC subsidiaries in Korea has increasingly become a critical topic of interest. Previously, the MNC subsidiaries in Korea, with limited information regarding their investment purpose, were excluded from the subsidiaries in Korea listed in INSC’s dataset. Secondly, subsidiaries operating for less than 5 years were excluded from the dataset to capture the long-term performance of MNC subsidiaries. Finally, subsidiary units with definite contact information were selected.

Structured questionnaire items were prepared as the primary data source. The respondents were employees having general work experiences at the subsidiary level since this research aimed to investigate the relationship between ESG and the performance of MNC subsidiaries located in Korea. Participants, targeted employees of MNC subsidiaries, were required to complete our questionnaire concerning their business activities and other key variables such as ESG and their company backgrounds. Before sending the questionnaire, items for the survey were developed in accordance with an inclusive literature review and consultation with researchers. Initially, questionnaires were responded to from the initial calls and follow-up e-mailing. After excluding data that had missing points and were not suitable for this research, the final data were collected.

### Variable Measurements

A survey instrument was developed to capture the four building blocks: ESG, financial performance, non-financial performance, and market-oriented organizational culture of MNC subsidiaries. Seven-point Likert-type scales were used for all measures, ranging from 1 = “strongly disagree” to 7 = “strongly agree.”

#### Environmental, Social, and Governance of the Subsidiary

Environmental, social, and governance were utilized as antecedents of financial and non-financial performance in this research. Specifically, we used the ESG measure developed by Zhou and Wang (2020). ESG was measured comprehensively by asking respondents to rate each of the items.

#### Financial Performance of Subsidiary

The financial performance was measured by aggregating items to embrace the long-term performance of a foreign subsidiary in the host country in different dimensions regarding their competitors in the market. Owing to the scarce reliable profitability data at the level of subsidiaries, a multi-dimensional construct that includes aspects such as profitability, productivity, and market share relative to peer organization which comes from a well-designed multi-item questionnaire, is thought to be suitable (Meyer et al., 2020).

#### Non-financial Performance of Subsidiary

As this research focused on the non-financial performance of subsidiaries and financial performance, the respondents were asked to answer questions of items regarding non-financial performance. The non-financial performance measures the satisfaction of customers and employees, and reputation links to the stakeholder interests.

#### Market-Oriented Organizational Culture

The market-oriented organizational culture was developed by Cameron and Quinn (2011). Their definition of market-oriented organizational culture includes the performance-oriented culture of the organization. The respondents were asked to answer questions concerning items as this research concentrated on the culture can affect the relationship between a subsidiary’s ESG and performance. These items include the organization’s overall environment in terms of specific results and the underlying culture that drives organizational success.

### Control Variables

Studies drawing on stakeholder and institutional perspectives have not accounted for the fact that firms are heterogeneous, with different resources and capabilities (Barney, 1991). However, this heterogeneity in firm attributes may influence the subsidiary performances. Consequently, we include subsidiary-level, MNC-level, and local market-level control variables to control their effects on subsidiary performance. For subsidiary-level control variables, we included previous performance, subsidiary autonomy, subsidiary age, and the industry of the subsidiary. The autonomy of the subsidiary measure was developed by O’Donnell (2000). Also, the industry of the subsidiary is proxied as dummies: 1 if the subsidiary is operating in the manufacturing industry and 0 otherwise. For the MNC-level control variables, we controlled for a parent equity share in the subsidiary and international strategies of the head office, HQ global orientation, and local responsiveness. Specifically, we used the HQ global orientation measure developed by Bartlett and Ghoshal (2002) and local responsiveness developed by Yang and Rivers (2009). Parent equity share was measured as dummies: 1 if the subsidiary’s type of foreign direct investment (FDI) is a greenfield investment, and 0 if the subsidiary’s type of FDI is a brownfield investment. For the local market-level control variables, we controlled for the subsidiary’s level of localization. Lastly, we used these control variables of ESG influence to measure their effect on the performance of subsidiaries.

### Validity and Reliability

The validity and reliability of measures were estimated. Content validity was ensured when developing the survey instrument through relevant literature reviews and in-depth interviews with employees of MNE subsidiaries. In addition, the discriminant validity, unidimensionality, and reliability were measured by factor analysis. The reliability of all constructs exceeded 0.70 in Cronbach’s alpha (Hinton et al., 2004). As a result, it was concluded that all measures exhibited an appropriate level of discriminant validity and unidimensionality.

Most of the variables, including independent and dependent variables in the research model, were measured by the answers of the same informants who completed the same questionnaire, which may bring on common method bias (Podsakoff et al., 2003). We conduct Harman’s single factor test employing principal component analysis to control common method bias and check for construct validity. This single factor explained the variation, which is less than the 50% threshold in latent constructs overall. Consequently, we conclude that the effect of the common method bias is hardly problematic in this study.

### Regression Method

This study used a regression model by adopting the generalized least squares (GLSs) procedure. GLS involves transforming the original variables into the converted variables which satisfy the OLS assumptions (Kmenta, 1971). The GLS procedure partitions error variance into three components – random error in space, random error in time, and random error not unique to space or time – and uses this information to draw efficient and unbiased parameter estimates (Sayrs, 1989). Furthermore, GLS assumes that the intercept is acquired from a normal distribution and is statistically independent of the explanatory variables in the model. GLS is used to capture the precise relationship between the variables considering both time and space by minimizing the error between variables. The STATA program’s xt family of commands was used to perform the analysis for this research.

We first explored which effect would be appropriate for our research between fixed effect and random effect using dummy variables of country-level when we analyzed the research model using the GLS procedure. We discarded the fixed-effects approach not only because some of our independent variables were remarkably stable over space for our countries but also because we lost a significant number of observations. In the case where the fixed-effects approach is not appropriate, a random-effects method can be used wherein the fixed effects are uncorrelated with the other independent variables (Cannella et al., 2008). A Hausman test (Hausman and Taylor, 1981) revealed no significant correlations between our independent variables and the country-level fixed effects. Another Hausman test is recommended if the preferred random effect *p*-value is greater than 5%, validating our choice of random-effects models in testing our hypotheses (Schaffer and Stillman, 2006).

## Empirical Results and Discussion

### Descriptive Statistics

Table 1 shows the characteristics distribution of the sample subsidiaries of MNCs. We can find the number of employees (more than 100–42.8%), CEO’s nationality (headquarters’ country 40.6% and Korea 50.7%), Industry classification (manufacturing 59.4%), types of investment (single investment 60.1%), types of business operation (manufacturing and sales 31.2%), and year of entry (after 2000 63%).

**TABLE 1:** Characteristics’ distribution of the sample subsidiaries of MNCs.

		Persons	%
Total respondent		138	100.0
Number of employees	Less than 30	50	36.2
	Between 31 and 99	26	18.8
	More than 100	59	42.8
	No answer	3	2.2
CEO’s nationality	Headquarters’ country	56	40.6
	Korea	70	50.7
	Other countries	12	8.7
Industry	Manufacturing	82	59.4
	Service	56	40.6
Types of investment	Single investment	83	60.1
	Joint investment	33	23.9
	Merge of local firms	11	8.0
	Others	11	8.0
Types of business operation	Manufacturing	20	14.5
	Sales	37	26.8
	Manufacturing and sales	43	31.2
	Service	38	27.5
Year of entry	Before 1999	51	37.0
	After 2000	87	63.0
Sales revenue of subsidiary firm in 2021	Less than U$45 million	77	55.8
	More than U$45 million	53	38.4
	No answer	8	5.8
Headquarters’ share of investment to subsidiary	Less than 100%	32	23.2
	100%	85	61.6
	No answer	21	15.2
Location of headquarter	North America	31	22.5
	Asia and Oceania	60	43.5
	Europe	47	34.1

Table 2 reports the descriptive statistics. The mean value of *FINANCIAL_PERFORMANCE*, *NON-FINANCIAL_PERFORMANCE*, and *SUBSIDIARY’S_ESG* is 4.59, 4.63, and 4.50, respectively, implying that the performance and ESG are above average. *MARKET-ORIENTED_ORG_CULTURE* is 4.93, which is much higher than average. The mean value of *SUBSIDIARY_AGE* is 20.86. Table 3 shows the Pearson correlation matrix. The correlation coefficients of the independent variables are less than 0.53, suggesting that there are no material problems of multicollinearity. *SUBSIDIARY’S_ESG* is significantly and positively correlated with *FINANCIAL_PERFORMANCE* (0.414, *p* < 0.01) and *NON-FINANCIAL_PERFORMANCE* (0.530, *p* < 0.01).

**TABLE 2:** Descriptive statistics.

Variable	*N*	Mean	p50	SD	p25	p75
*FINANCIAL_PERFORMANCE*	105	4.59	4.33	1.14	4.00	5.33
*NON-FINANCIAL_PERFORMANCE*	105	4.63	4.67	0.98	4.00	5.33
*SUBSIDIARY’S_ESG*	105	4.50	4.51	1.02	3.96	5.17
°*Environmental*	105	4.52	4.50	1.24	4.00	5.25
*°Social*	105	4.79	4.80	1.12	4.00	5.40
°*Governance*	105	4.18	4.25	1.34	3.38	5.00
*MARKET-ORIENTED_ORG_CULTURE*	105	4.93	5.00	0.84	4.33	5.67
*SALES*	105	5.78	5.66	1.91	4.47	7.23
*PARENT EQUITY_SHARE*	105	0.38	0.00	0.49	0.00	1.00
*SUBSIDIARY_AGE*	105	20.86	19.00	12.05	12.00	26.00
*HQ_GLOBAL_ORIENTATION*	105	4.85	5.00	1.32	4.00	6.00
*LOCAL_RESPONSIVENESS*	105	4.84	5.00	1.12	4.00	5.50
*SUBSIDIARY’S_LEVEL_OF_ LOCALIZATION*	105	4.77	4.75	1.08	4.00	5.50
*SUBSIDIARY_AUTONOMY*	105	4.09	4.00	1.15	3.29	4.86
*INDUSTRY*	105	0.35	0.00	0.48	0.00	1.00

**TABLE 3:** Correlation matrix (*n* = 105).

	1	2	3	4	5	6	7	8	9	10	11
*1. FINANCIAL_PERFORMANCE*	1										
*2. NON-FINANCIAL_PERFORMANCE*	0.687***										
*3. SUBSIDIARY’S_ESG*	0.414***	0.530***									
*4. MARKET-ORIENTED_ORGANIZATIONAL_ CULTURE*	0.308**	0.461***	0.482***								
*5. SALES*	0.279**	0.241*	0.450***	0.238*							
*6. PARENT_EQUITY_ SHARE*	–0.088	0.004	–0.066	–0.039	–0.160						
*7. SUBSIDIARY_AGE*	0.023	–0.007	0.115	–0.107	0.429***	–0.086					
*8. HQ GLOBAL_ORIENTATION*	0.351***	0.466***	0.235*	0.366***	0.108	0.024	0.025				
*9. LOCAL_RESPONSIVENESS*	0.429***	0.504***	0.419***	0.446***	0.217*	–0.018	0.046	0.436***			
*10. SUBSIDIARY’S_LEVEL_OF_ LOCALIZATION*	0.172^†^	0.181^†^	0.261**	0.182^†^	0.176^†^	0.125	–0.018	0.031	0.394***		
*11. SUBSIDIARY_AUTONOMY*	0.216*	0.177^†^	0.205*	0.225*	0.122	0.144	0.035	0.058	0.347***	0.530***	
*12. INDUSTRY*	0.071	–0.012	–0.061	0.002	−0.205*	0.037	−257**	–0.089	–0.063	–0.140	–0.069

^†^p < 0.10, *p < 0.05, **p < 0.01, ***p < 0.001.

*FINANCIAL_PERFORMANCE = mean value of the survey data of comprehensive performance, sales growth rate, market share ratio, and operating profit; NON-FINANCIAL_PERFORMANCE = mean value of the survey data of customer satisfaction, employee satisfaction, and reputation and image; SUBSIDIARY’S_ESG = environmental, social, and governance (ESG) of multinational corporation’s subsidiary, the mean value of environmental, social, and governance; MARKET-ORIENTED_ORG_CULTURE = subsidiary firm’s market-oriented organizational culture; SALES = natural logarithm of sales revenue for the fiscal year: PARENT EQUITY_SHARE = parent firm’s equity share; SUBSIDIARY_AGE = subsidiary firm’s age; HQ_GLOBAL_ORIENTATION = headquarters’ global orientation; LOCAL_RESPONSIVENESS = subsidiary firm’s local responsiveness; SUBSIDIARY’S_LEVEL_OF_LOCALIZATION = subsidiary firm’s level of localization; SUBSIDIARY_AUTONOMY = subsidiary firm’s autonomy; and INDUSTRY = industry dummy taking 1 if the firms belong to the Manufacturing, 0 otherwise.*

In addition, each value of the variance inflation factor (VIF) was calculated. The VIFs ranged between 1.08 and 1.76 with an average of 1.46, suggesting no material problems of multicollinearity in the analysis (Chatterjee and Hadi, 2013). The result reported in Table 3 implies that the relationships among key variables—subsidiary’s ESG and financial performance and subsidiary’s ESG and non-financial performance—are precisely in line with the directions of the hypotheses established. Also, Tables 4, 5 report the regression results. In Tables 4, 5, the subsidiary’s ESG performance is the independent variable, and financial and non-financial performance are the dependent variables. We will examine each model in turn as we consider the hypotheses.

**TABLE 4:** The effects of subsidiary’s ESG on financial and non-financial performance.

	Dependent variable			
Independent variables	*FINANCIAL_PERFORMANCE*		*NON-FINANCIAL_PERFORMANCE*	
	Model 1	Model 2	Model 3	Model 4
*SUBSIDIARY’S_ESG*		0.233* (0.115)		0.350*** (0.089)
*SALES*	0.137* (0.060)	0.087 (0.064)	0.096* (0.049)	0.022 (0.049)
*PARENT_EQUITY_SHARE*	−0.180 (0.208)	−0.171 (0.205)	0.040 (0.170)	0.054 (0.159)
*SUBSIDIARY_AGE*	−0.005 (0.009)	−0.004 (0.009)	−0.008 (0.008)	−0.006 (0.007)
*HQ_GLOBAL_ORIENTATION*	0.195* (0.085)	0.181* (0.084)	0.229** (0.069)	0.207** (0.065)
*LOCAL_RESPONSIVENESS*	0.261* (0.111)	0.205* (0.112)	0.288** (0.090)	0.204* (0.087)
*SUBSIDIARY’S_LEVEL_OF_LOCALIZATION*	−0.003 (0.114)	−0.021 (0.113)	0.001 (0.093)	−0.025 (0.087)
*SUBSIDIARY_AUTONOMY*	0.106 (0.103)	0.102 (0.102)	0.016 (0.084)	0.009 (0.079)
*INDUSTRY*	0.354 (0.216)	0.333 (0.212)	0.104 (0.176)	0.072 (0.164)
*INTERCEPT*	1.238^†^ (0.659)	0.899 (0.671)	1.608** (0.538)	1.098* (0.518)
Wald Chi-Square	39.38***	44.70***	53.64***	77.08***
R^2^	0.29	0.32	0.36	0.45
Number of samples	105	105	105	105

^†^p < 0.10, *p < 0.05, ^**^p < 0.01, ^***^p < 0.001.

*FINANCIAL_PERFORMANCE = mean value of the survey data of comprehensive performance, sales growth rate, market share ratio, and operating profit; NON-FINANCIAL_PERFORMANCE = mean value of the survey data of customer satisfaction, employee satisfaction, and reputation and image; SUBSIDIARY’S_ESG = Environmental, Social, and Governance (ESG) of Multinational corporation’s subsidiary, the mean value of environmental, social, and governance; SALES = natural logarithm of sales revenue for the fiscal year: PARENT EQUITY_SHARE = parent firm’s equity share; SUBSIDIARY_AGE = subsidiary firm’s age; HQ_GLOBAL_ORIENTATION = headquarters’ global orientation; LOCAL_RESPONSIVENESS = subsidiary firm’s local responsiveness; SUBSIDIARY’S_LEVEL_OF_LOCALIZATION = subsidiary firm’s level of localization; SUBSIDIARY_AUTONOMY = subsidiary firm’s autonomy; and INDUSTRY = industry dummy taking 1 if the firms belong to the Manufacturing, 0 otherwise.*

**TABLE 5:** The moderate effect of market-oriented organizational culture.

	Dependent variable			
Independent variables	*FINANCIAL_PERFORMANCE*		*NON-FINANCIAL_PERFORMANCE*	
	Model 1	Model 2	Model 3	Model 4
*SUBSIDIARY’S_ESG*	0.233* (0.115)	1.474* (0.594)	0.350*** (0.089)	0.321 (0.467)
*MARKET-ORIENTED_ORG_CULTURE*		1.075* (0.549)		0.123 (0.432)
*SUBSIDIARY’S_ESG×MARKET-ORIENTED_ORG_CULTURE*		−0.239* (0.113)		−0.001 (0.089)
*SALES*	0.087 (0.064)	0.065 (0.064)	0.022 (0.049)	0.016 (0.051)
*PARENT_EQUITY_SHARE*	−0.171 (0.205)	−0.125 (0.204)	0.054 (0.159)	0.059 (0.160)
*SUBSIDIARY_AGE*	−0.004 (0.009)	−0.005 (0.009)	−0.006 (0.007)	−0.004 (0.007)
*HQ_GLOBAL_ORIENTATION*	0.181* (0.084)	0.164* (0.085)	0.207** (0.065)	0.192** (0.067)
*LOCAL_RESPONSIVENESS*	0.205* (0.112)	0.205* (0.113)	0.204* (0.087)	0.185* (0.089)
*SUBSIDIARY’S_LEVEL_OF_LOCALIZATION*	−0.021 (0.113)	−0.030 (0.111)	−0.025 (0.087)	−0.020 (0.088)
*SUBSIDIARY_AUTONOMY*	0.102 (0.102)	0.112 (0.101)	0.009 (0.079)	0.001 (0.079)
*INDUSTRY*	0.333 (0.212)	0.286 (0.211)	0.072 (0.164)	0.067 (0.166)
*INTERCEPT*	0.899 (0.671)	−4.354* (2.641)	1.098* (0.518)	0.804 (2.078)
Wald Chi-Square	44.70***	50.48***	77.08***	77.55***
R^2^	0.32	0.35	0.45	0.45
Number of samples	105	105	105	105

^†^p < 0.10, *p < 0.05, ^**^p < 0.01, ^***^p < 0.001.

*MARKET-ORIENTED_ORG_CULTURE = subsidiary firm’s market-oriented organizational culture.*

### Regression Analyses

Table 4 provides the test results for Hypothesis 1 and 2. Model 1 of Table 4 is a baseline model that includes only control variables such as previous performance, parent equity share, subsidiary age, HQ global orientation, local responsiveness, subsidiary’s level of localization, subsidiary autonomy, and industry. First, we included ESG separately in the estimation model. Model 2 reveals the positive effect of subsidiary’s ESG on the financial performance (*b* = 0.233, *p* < 0.05), where Model 4 shows the positive effect of subsidiary’s ESG on the non-financial performance (*b* = 0.350, *p* < 0.001). These results provide strong evidence to support Hypothesis 1 and 2 simultaneously. The ESG of MNC subsidiaries operating in Korea positively influences financial performance and non-financial performance. Additionally, we conclude that the regression models have reasonable explanatory power since the *R*^2^ is greater than 0.29.

The results in Table 5 show the moderating effect of market-oriented organizational culture on the relationship between a subsidiary’s ESG and performance. Specifically, Model 1 and Model 3 of Table 5 are baseline models consisting of only control variables, including ESG influencing financial and non-financial performance. Then, we entered the interaction term between ESG and market-oriented organizational culture in the estimation model. Model 2 reveals that this interaction term is negative and significant (*b* = −0.239, *p* < 0.05), suggesting that the relationship between a subsidiary’s ESG and financial performance is weakened when subsidiaries in Korea have a market-oriented organizational culture. Thus, Hypothesis 3 is supported. On the other hand, the interaction term between ESG and market-oriented organizational culture is insignificant in Model 4. Therefore, Hypothesis 4 is not supported.

## Discussion and Conclusion

This study analyzed how much ESG management performed by a Korean subsidiary of MNCs contributes to the subsidiaries’ performance. In addition, we examined how market-oriented organizational culture moderates the relationship between ESG activities and performance. The relationship between ESG and performance was considered an extension of existing CSR research theories, including institutional duality theory and resource-based theory. Moreover, the moderating effect of market-oriented organizational culture on the relationship was designed as a research model. Data were collected by surveying Korean subsidiaries of MNCs, and hypotheses were verified through regression analyses.

The analyses results are summarized as follows. First, ESG management has a significantly positive relationship with financial and non-financial performance. These results are consistent with prior studies (Waddock and Graves, 1997; Godfrey et al., 2009) that corporate activities related to social responsibility positively affect business performance.

Also, we find that the market-oriented organizational culture of Korean subsidiaries of MNCs had a significantly negative effect on the relationship between ESG and financial performance.

The theoretical implications of this study are as follows. First, our study provides empirical evidence that the ESG activities of subsidiaries of MNCs have a positive effect on firm performance. Why can ESG activities of overseas subsidiaries have a positive effect on business performance? We can explain the reason through the resource-based theory and the stakeholder theory. Second, the empirical results of the moderating effect of market-oriented organizational culture can be explained through institutional duality along with strategic fit. Since the institutional duality of MNCs broadens the spectrum of strategic choices, certain strategies chosen by subsidiaries may not be a good fit for each other. In conclusion, the results of our empirical analysis suggest that a market-oriented organizational culture and ESG management do not present a strategic fit in the Korean market.

The following practical implications can be drawn from the results of this study. First, ESG management of Korean subsidiaries of MNCs is a driving force that can overcome the liability of foreignness and improve the subsidiaries’ financial and non-financial performance. Therefore, MNCs should strategically initiate and implement ESG activities for the subsidiary that are intentionally tailored to meet the needs of its immediate business environment, thereby directly enhancing the subsidiary’s performance and overall value.

Second, subsidiaries’ market-oriented organizational culture fosters an environment where subsidiaries focus on short-term performance. Consequently, ESG is more likely to be perceived as a cost or an unavoidable procedure rather than an immediate benefit to a subsidiary in fierce competition. Because ESG focuses on long-term rather than short-term performance, it contrasts with the short-term performance orientation driven by a market-oriented organizational culture. This suggests that the subsidiaries’ market-oriented organizational culture can become an obstacle to ESG activities’ path to positive and tangible outcomes. Establishing an organizational culture is critical to the success of the firm. Thus, it is necessary to pay more attention to the fact that ESG management, which emphasizes procedures, is the driving force leading to performance.

Although this study draws meaningful conclusions through empirical analysis of subsidiaries of MNCs in Korea on the relationship between ESG management and the performance of MNCs, our study has the following limitations. First, we made efforts to categorize and conceptualize the activities of ESG management of Korean subsidiaries of MNCs. However, there are difficulties in systematizing them to gain consensus due to the nature of the concept of ESG management.

In addition, since we attempted to verify a hypothesis through survey data of overseas subsidiaries, the homogeneity of the survey respondents was not secured, so there is a possibility that the respondents responded in a situation where they did not fully understand the concept and type of ESG management. Moreover, it is possible that the respondents are not directly involved with the ESG-related department or responded based on insufficient information about the parent company and strategic direction from the subsidiary’s perspective.

Despite these limitations, this study differentiated it from existing studies and conducted an empirical analysis of the ESG activities and performance of subsidiaries of MNCs through a questionnaire survey. Through this, we confirmed previous studies’ results that subsidiaries’ ESG has a positive effect on financial and non-financial performance. In addition, we find that a market-oriented corporate culture, which emphasizes short-term performance, weakens the positive association between ESG and firm performance. This could be due to the long-term orientation of ESG activities. Therefore, the results of this study are significant in that it provides valuable information that practitioners can refer to in their work when pursuing business strategies that maximize a firm long-term value.

## Data Availability Statement

The original contributions presented in the study are included in the article/supplementary material, further inquiries can be directed to the corresponding author.

## Author Contributions

All authors listed have made a substantial, direct, and intellectual contribution to the work, and approved it for publication.

## Conflict of Interest

The authors declare that the research was conducted in the absence of any commercial or financial relationships that could be construed as a potential conflict of interest.

## Publisher’s Note

All claims expressed in this article are solely those of the authors and do not necessarily represent those of their affiliated organizations, or those of the publisher, the editors and the reviewers. Any product that may be evaluated in this article, or claim that may be made by its manufacturer, is not guaranteed or endorsed by the publisher.

## Appendix

**TABLE A1:** Detailed items of ESG.

*SUBSIDIARY’S_ESG*: environmental	The degree of (1) environmental protection training and education, (2) investment in environmental protection, (3) energy consumption water consumption, and (4) R&D and application of environmental protection technology equipment
*SUBSIDIARY’S_ESG*: social	The degree of (1) gender equality in the subsidiary, (2) employees participating in trade unions, (3) protection of employees’ personal privacy and guarantee of paid vacation days per year, (4) support for education, local governments, and NGOs, and (5) support for employee volunteer systems and employee volunteer activities
*SUBSIDIARY’S_ESG*: governance	The degree of (1) CSR planning or annual planning, (2) social responsibility organization system, (3) stakeholder identification, (4) stakeholder expectations and corporate response measures, (5) whether to issue a social responsibility report, (6) whether a CSR column exists on the official website, (7) social responsibility activities that involve senior leaders, and (8) social responsibility activities that involve employees
